# Fibrin and Collagen Differentially but Synergistically Regulate Sprout Angiogenesis of Human Dermal Microvascular Endothelial Cells in 3-Dimensional Matrix

**DOI:** 10.1155/2013/231279

**Published:** 2013-04-30

**Authors:** Xiaodong Feng, Marcia G. Tonnesen, Shaker A. Mousa, Richard A. F. Clark

**Affiliations:** ^1^Department of Clinical and Administrative Sciences, California Northstate University College of Pharmacy, Rancho Cordova, CA 95670, USA; ^2^Department of Dermatology, School of Medicine, State University of New York at Stony Brook, Stony Brook, NY 11794, USA; ^3^Dermatology Section, Veterans Affairs Medical Center, Northport, NY 11768, USA; ^4^Pharmaceutical Research Institute, Albany College of Pharmacy and Health Sciences, Albany, NY 12208, USA; ^5^Center for Tissue Engineering, State University of New York at Stony Brook, Stony Brook, NY 11794, USA

## Abstract

Angiogenesis is a highly regulated event involving complex, dynamic interactions between microvascular endothelial cells and extracellular matrix (ECM) proteins. Alteration of ECM composition and architecture is a hallmark feature of wound clot and tumor stroma. We previously reported that during angiogenesis, endothelial cell responses to growth factors are modulated by the compositional and mechanical properties of a surrounding three-dimensional (3D) extracellular matrix (ECM) that is dominated by either cross-linked fibrin or type I collagen. However, the role of 3D ECM in the regulation of angiogenesis associated with wound healing and tumor growth is not well defined. This study investigates the correlation of sprout angiogenesis and ECM microenvironment using in vivo and in vitro 3D angiogenesis models. It demonstrates that fibrin and type I collagen 3D matrices differentially but synergistically regulate sprout angiogenesis. Thus blocking both integrin alpha v beta 3 and integrin alpha 2 beta 1 might be a novel strategy to synergistically block sprout angiogenesis in solid tumors.

## 1. Introduction

Angiogenesis, the development of new blood vessels from preexisting vessels, is critical for a wide array of complex normal and pathological processes including morphogenesis, wound healing, and tumor growth [1]. Under normal physiologic conditions, angiogenesis is well controlled by the local balance between endogenous angiogenesis stimulators and angiogenesis inhibitors, although the regulatory mechanism is still not clearly defined. Sustained tumor angiogenesis is one of the hallmark features of solid tumor development. It is essential for tumor development and tumor metastasis. Almost four decades ago Dr. Judah Folkman pioneered the strategy of stopping tumor growth and metastasis by blocking tumor angiogenesis. With the 2004 FDA approval of bevacizumab (Avastin), a humanized monoclonal antibody against vascular endothelial growth factor (VEGF), to treat metastatic colorectal cancer in combination with 5-fluorouracil (5-FU), antiangiogenesis therapy has emerged as an essential new strategy for cancer treatment [2].

 Angiogenesis is a highly regulated event that involves complex, dynamic interactions between microvascular endothelial cells and ECM proteins. In developing capillary sprouts, endothelial cells digest the surrounding extracellular matrix (ECM) and invade the matrix as a cylindrical aggregate of cells. These events clearly require an integrated response of endothelial cells to angiogenic factors and ECM proteins [3]. Alteration of ECM composition and architecture is a hallmark of wound clot and tumor stroma. ECM matrices induce multiple dynamic interactions with endothelial cells and stimulate the transduction of signals by cross-linking integrin receptors on endothelial cells. Initially viewed as merely a physical barrier, the ECM is now recognized as having a profound effect on the angiogenic phenotype. However, the integrated regulatory mechanism of microvascular endothelial cell response to ECM and angiogenic factors is poorly defined [4, 5]. In addition, numerous evidences indicate that the in vitro cellular regulations of many cell types in 2D environment are significantly different than those of cells in 3D environment. Since 3D environment is more close to the in vivo microenvironment of cell functions, it suggests that reproducible and quantifiable in vitro 3D assays play an important role to study the regulation of cellular behaviors during physiological and pathological processes [6].

Fibrin and type I collagen are two major components of extracellular matrix microenvironment. Fibrin deposition is commonly observed in angiogenesis associated with wound healing and tumor growth. It has been reported that fibrin enhances angiogenesis of wound healing in vitro [7] and in vivo [8]. In contrast, type I collagen is a major component of normal dermis which has minimal angiogenesis activities, although some in vitro studies demonstrate that type I collagen gel supports angiogenesis as well as fibrin gel. The results of these in vitro studies are not consistent with the in vivo data reported by Dvorak et al. that fibrin but not type I collagen induces angiogenesis in vivo [8].

Integrin alpha v beta 3 is the receptor for fibrin matrix. Expression of integrin alpha v beta 3 is one of the hallmark features of sprout angiogenesis. Remarkably, integrin beta 3 expression was highly upregulated in vascular endothelial cells found in fibrin rich but not in collagen rich matrix environment in vivo and in vitro. We recently demonstrated that fibrin and collagen differentially regulated integrin expression in human dermal microvascular endothelial cells (HDMEC) [4] and in human dermal fibroblasts [9]. In particular, fibrin, but not collagen, increased the expression of integrin alpha v beta 3 in HDMEC [4]. Since integrin alpha v beta 3 expression is differentially regulated by ECM and it is required for an angiogenic response to certain angiogenic factors, such as VEGF and bFGF [10], we hypothesized that fibrin and collagen differentially regulate angiogenesis. 

Angiogenesis is a tightly regulated event, which visually includes endothelial invasion, migration, capillary tube formation, and capillary network formation. It is essential to have a reproducible and quantifiable in vitro assay of human sprout angiogenesis to investigate the integrated response of human microvascular endothelial cells to angiogenic factors and 3D ECM. Using a modified microcarrier-based 3D angiogenesis assay [11–13], we demonstrated in vitro that fibrin and collagen differentially regulate sprout angiogenesis. Our in vitro data also indicated that fibrin was essential for sprout angiogenesis of human microvascular endothelial cells in response to angiogenic factors. This was consistent with our in vivo data that there was a close correlation between fibrin presence and sprout angiogenesis occurrence in porcine wound. Inversely, when fibrin was almost totally replaced by collagen, sprout angiogenesis regressed. In addition, for the first time we demonstrated that integrin alpha v beta 3 (receptor for fibrin) and integrin alpha 2 beta 1 (receptor for collagen) differentially but synergistically regulate sprout angiogenesis.

## 2. Materials and Methods

### 2.1. Materials

Gelatin-coated microcarrier beads (Cytodex-3) were purchased from Pharmacia (Uppsala, Sweden). Dimethyl dichlorosilane, aprotinin, dibutyryl cyclic AMP, hydrocortisone, trypsin, soybean trypsin inhibitor, and EDTA were obtained from Sigma Chemical Co. (St. Louis, MO, USA). Cyclic RGD, Gly-Pen-Gly-Arg-AspPro-Cys-Ala (GpenGRGDSPCA), Vitronectin specific GPenGRGDSPCA peptides (RGD (VN)), and inactive sham control peptide, Gly-Arg-Ala-Asp-Ser-Pro (GRADSP), from Life Technologies (Carlsbad, CA, USA). Endothelial cell basal medium (EBM), endothelial cell growth medium bulletkit-2 (EGM-2 Bulletkit), bovine brain extract, and epidermal growth factor were obtained from Clonetics Corp. (San Diego, CA, USA). Normal human serum was obtained from BioWhittaker, Inc. (Walkersville, MD, USA). Vascular endothelial cell growth factor (VEGF) was purchased from Peprotech (Rocky Hill, NJ, USA). Basic fibroblast growth factor (bFGF) was obtained from Scios Nova, Inc. (Mountainvale, CA, USA). Human thrombin was obtained from Calbiochem (San Diego, CA, USA). Propidium iodide (PI) was obtained from Molecular Probes (Eugene, OR, USA). Integrin alpha 2 beta 1 blocking monoclonal antibody MAB1998 was obtained from Chemicon. Disintegrin ELP12 was a kindly gift from Cezary Marcinkiewicz of Temple University, PA, USA. Echistatin was obtained from Sigma Chemical Co. (St. Louis, MO, USA).

### 2.2. Cell Culture

 Human dermal microvascular endothelial cells (HDMEC) were isolated from human neonatal foreskins as previously reported [4]. Briefly, after initial harvest from minced trypsinized human foreskins, microvascular endothelial cells were further purified on a Percoll density gradient. HDMEC were cultured on collagen type 1 coated tissue culture flasks in EGM (endothelial cell growth medium) consisting of EBM supplemented with 10 ng/mL epidermal growth factor, 0.4% bovine brain extract, 17.5 microgram/mL dibutyryl cyclic AMP, and 1 microg/mL hydrocortisone in the presence of 30% normal human serum. Endothelial cell cultures were characterized and determined to be >99% pure on the basis of formation of typical cobblestone monolayers in culture, positive immunostaining for factor VIII-related antigen, and selective uptake of acetylated low density lipoprotein. All experiments were done with HDMEC below passage 8. 

### 2.3. Preparation of Endothelial Cell-Loaded Microcarrier Beads (EC-Beads)

 Gelatin-coated cytodex-3 microcarrier beads were prepared as described by the manufacturer. Approximately 80,000 sterile microcarrier beads were washed, resuspended in EGM, and added to approximately 4.5 million endothelial cells (HDMEC). The beads and cells were mixed by gentle swirling, incubated at 37°C for 6 hr, and then rotated for 24–36 hr on an orbital mixer in a 37°C oven to generate endothelial cell-loaded microcarrier beads (EC-beads).

### 2.4. Cell Migration and Capillary Sprout Formation in Fibrin Gels and Type I Collagen Gels

**Figure 1:**
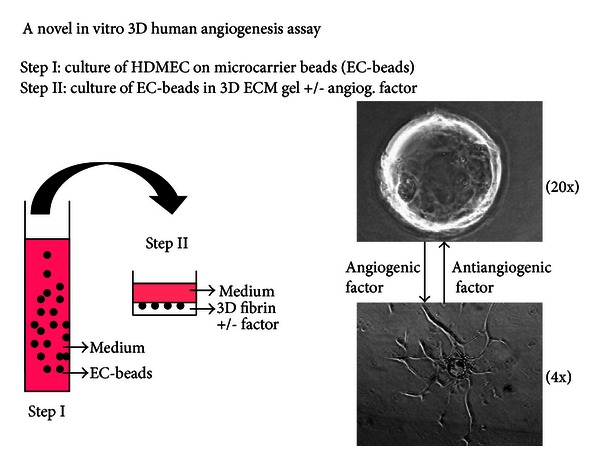
In vitro three-dimensional human angiogenesis model for assaying cell invasion, migration, and sprout angiogenesis formation of human dermal microvascular endothelial cells (HDMEC) (a). Step I: HDMEC are cultured on the surface of microcarrier beads to generate EC-beads. Step II: EC-beads are embedded in fibrin gel, collagen gel, or fibrin/collagen gel, with or without the presence of angiogenesis factor.

 A microcarrier in vitro angiogenesis assay previously designed to investigate bovine pulmonary artery endothelial cell angiogenic behavior in bovine fibrin gels [11–13] was modified for the study of human microvascular endothelial cell angiogenesis in different ECM environment (Figure 1(a)). Briefly, human fibrinogen, isolated as previously described [4], was dissolved in M199 medium at a concentration of 1 mg/mL (pH 7.4) and sterilized by filtering through a 0.22-micron filter. Pepsin-solubilized bovine dermal collagen dissolved in 0.012 M HCl was 99.9% pure containing 95–98% type I collagen and 2–5% type III collagen (Vitrogen 100, Collagen Biomaterials, Palo Alto, CA, USA). An isotonic 1.5 mg/mL collagen solution was prepared by mixing sterile Vitrogen 100 in 5X M199 medium and distilled water. pH was adjusted to 7.4 by 1 N NaOH. In certain experiments, angiogenic stimulators and/or inhibitors, such as VEGF, bFGF, RGD peptides, ELP12, and Echistatin, were added to the fibrinogen or collagen solutions (Figure 1). The angiogenic response was monitored visually and recorded by video image capture. Specifically, capillary sprout formation was observed and recorded with a Nikon Diaphot-TMD inverted microscope (Nikon Inc., Melville, NY, USA), equipped with an incubator housing with a Nikon NP-2 thermostat and Sheldon #2004 carbon dioxide flow mixer. The microscope was directly interfaced to a video system consisting of a Dage-MTI CCD-72S video camera and Sony 12′′ PVM-122 video monitor linked to a Macintosh G3 computer. The images were captured at various magnifications using Adobe Photoshop. The effect of angiogenic factors on sprout angiogenesis was quantified visually by determining the number and percent of EC-beads with capillary sprouts. One hundred beads (five to six random low power fields) in each of triplicate wells were counted for each experimental condition. All experiments were repeated at least three times. To locate the nucleus of HDMEC, the fibrin or collagen gel was fixed by methanol/acetone (1 : 1) and stained by 0.001% PI.

### 2.5. Porcine Cutaneous Wounds and Immunofluorescence Staining

Porcine cutaneous wounds were harvested at various times and then immunoprobed for expression of integrin receptors as previously described [14]. Briefly, full-thickness wounds were made with an 8 mm punch on the backs of White Yorkshire pigs and harvested at the times indicated. Specimens were bisected; one half was fixed in formalin and stained with Masson trichrome; the other half was frozen in liquid nitrogen for immunofluorescence studies. Antilaminin antibodies that were conjugated with biotin were used to identify wound vasculature. All antibodies were used at dilutions that gave maximal specific fluorescence and minimal background fluorescence on frozen tissue specimens. Bound antibody was detected by the avidin-biotin-complex (ABC) technique. Stained specimens were observed and photographed using a Nikon Microphot FXA epifluorescence microscope equipped with a Nikon FX-35DX 35 mm camera.

### 2.6. Confocal Microscopy

Confocal microscopy was done at the University Microscopy Imaging Center, Health Sciences Center, SUNY at Stony Brook, to confirm that sprouts emanating from the EC-beads formed tubes and to investigate the expression of integrin alpha v beta 3 on endothelial surface in 3D ECM. For these studies EC-beads (150) were suspended in a three-dimensional fibrin gel with VEGF (30 ng/mL) and bFGF (25 ng/mL) in a 4-well Lab-Tek Chambered Coverglass and incubated for 5 days. For experiments of tube formation, samples were washed in 2XPBS and then fixed in 2% paraformaldehyde. With the kind assistance of David Colflesh at the University Microscopy Imaging Center, images were sequentially obtained (1 micron contiguous tangential cross sections) using a Noran laser scanning confocal system (Odyssey; Noran Instruments Inc., Middleton, WI, USA) attached to a Nikon Diaphot-TMD microscope. A Silicon Graphics Iris workstation was used for processing digitized micrographs and assembling three-dimensional renditions from confocal images using Voxelview software (Voxelview; Vital Images). For experiments of immunostaining, 23C6, a monoclonal antibody to integrin beta 3, was used. 

## 3. Results

### 3.1. Positive Correlation of Fibrin and Collagen with Angiogenesis Sprouting and Regression in Wound Healing

**Figure 2:**
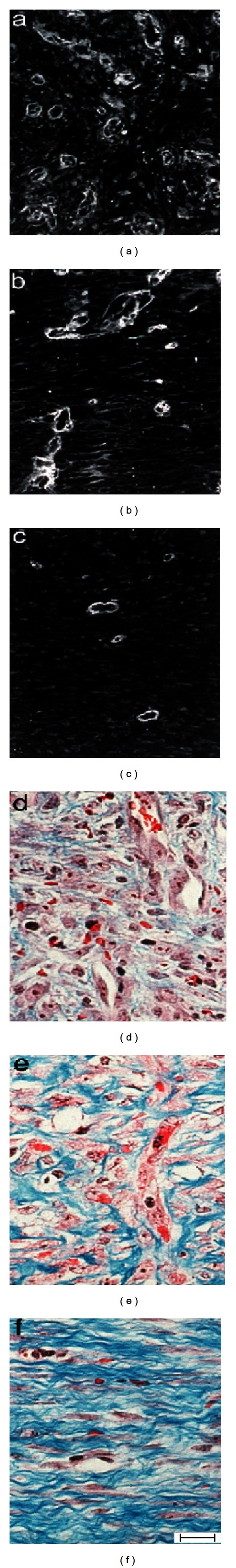
Angiogenic blood vessels in early granulation tissue mature and then regress as wound repair progresses. Porcine wounds at 5 days (a, d), 7 days (b, e), and 10 days (c, f) were stained with Masson trichrome (d, e, f) and antibody to laminin (a, b, c). At 5 days (a, d), the wound space is almost filled with granulation tissue, rich in newly forming microvessels. At 7 days (b, e), the neoepidermis has completely formed, the granulation tissue has organized, and the neovessels have matured and assumed a vertical orientation. At 10 days (c, f), wound contraction is underway, and blood vessel regression is apparent. Bar = 80 micron in (a, b, c) and 50 micron in (d, e, f).

 To understand the relationship between angiogenesis and different ECM components during granulation tissue formation of wound repair, we analyzed tissue specimens from 5-, 7-, and 10-day porcine wounds (Figure 2). The 5-day wounds are mainly composed of a fibrin-rich provisional matrix, whereas 7-day wounds have a substantial organized collagen fiber network, and 10-day wounds have developed a compacted contracted collagen scar [15, 16]. Staining of 5-, 7-, and 10-day wound specimens with Masson trichrome (Figures 2(d), 2(e), and 2(f)) and with antilaminin (Figures 2(a), 2(b), and 2(c)) revealed that the fibrin-rich early granulation tissue in 5-day wounds is filled with newly formed vessels (Figures 2(a) and 2(d)). These neovessels consistently stained weakly for laminin, most likely as a result of blood vessel immaturity. Such weak staining for laminin in immature blood vessels was previously observed by us in the microvasculature of human fetal skin [15]. By 7 days, the maturing blood vessels form an organized vertical array as collagen accumulates in the wound ECM (Figures 2(b) and 2(e). At 10 days, as the collagen bundles thicken to produce scar, many blood vessels are regressing (Figures 2(c) and 2(f)). There is a close correlation between fibrin presence and sprout angiogenesis occurrence in porcine wound. Sprout angiogenesis mainly occurred in day 5 when wound clot was mainly filled with fibrin (Figures 2(a) and 2(d)). Inversely, when fibrin was almost totally replaced by collagen on day 10, sprout angiogenesis regressed (Figures 2(c) and 2(f)). Thus during wound repair in vivo, the angiogenic neovessels in early granulation tissue invade the fibrin clot as capillary sprouts, mature, and then regress as fibrin is replaced by collagen in the wound space. 

### 3.2. 3D Fibrin Matrices with Angiogenic Factors Induce Sprout Angiogenesis of HDMEC In Vitro

**Figure 3:**
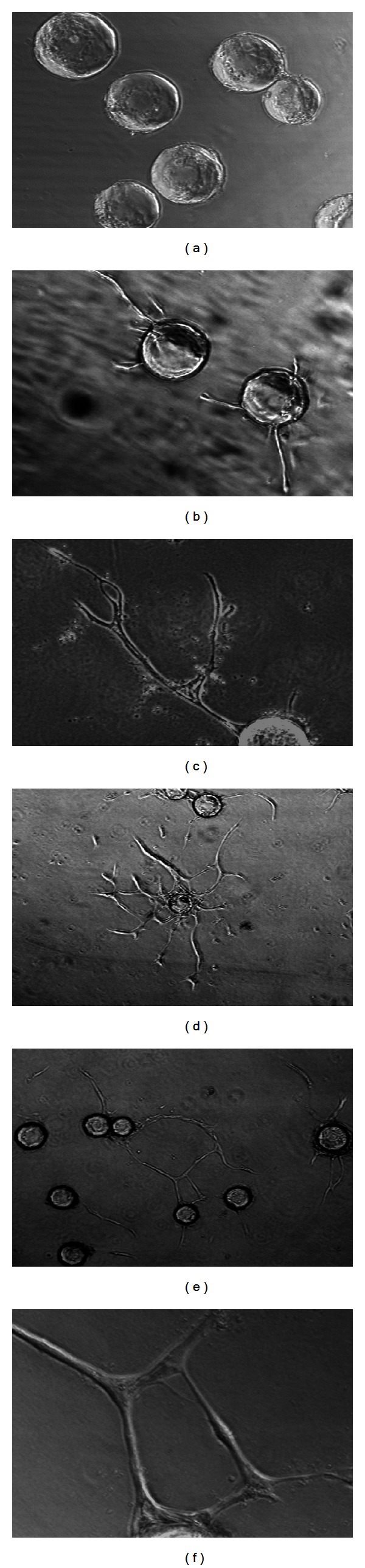
Formation of angiogenic sprouts and capillary tube-like structures by HDMEC in fibrin stimulated by VEGF (30 ng/mL) and bFGF (25 ng/mL). (a) In control fibrin gel without addition of angiogenic factors, no significant sprout formation occurred after 48 hr. (b) In the presence of VEGF (30 ng/mL) and bFGF (25 ng/mL), HDMEC formed angiogenic sprouts, and invaded and migrated into the fibrin gel within 48 hr. (c) By 5 days, VEGF and bFGF stimulated HDMEC forming branching capillary tube-like structures in the fibrin gel. (d) Formation of local capillary arcades and networks by HDMEC in fibrin with VEGF and bFGF for 5 days. (e) Formation of wild capillary networks by HDMEC in fibrin with VEGF and bFGF for 5 days. (f) Higher magnification of a typical capillary network illustrates that networks formed as a result of branching and fusion of capillary tubes from neighboring beads. Magnification: (a), (b), and (c) 100x, (d) 40x, (e) 40x, and (f) 200x.

**Figure 4:**
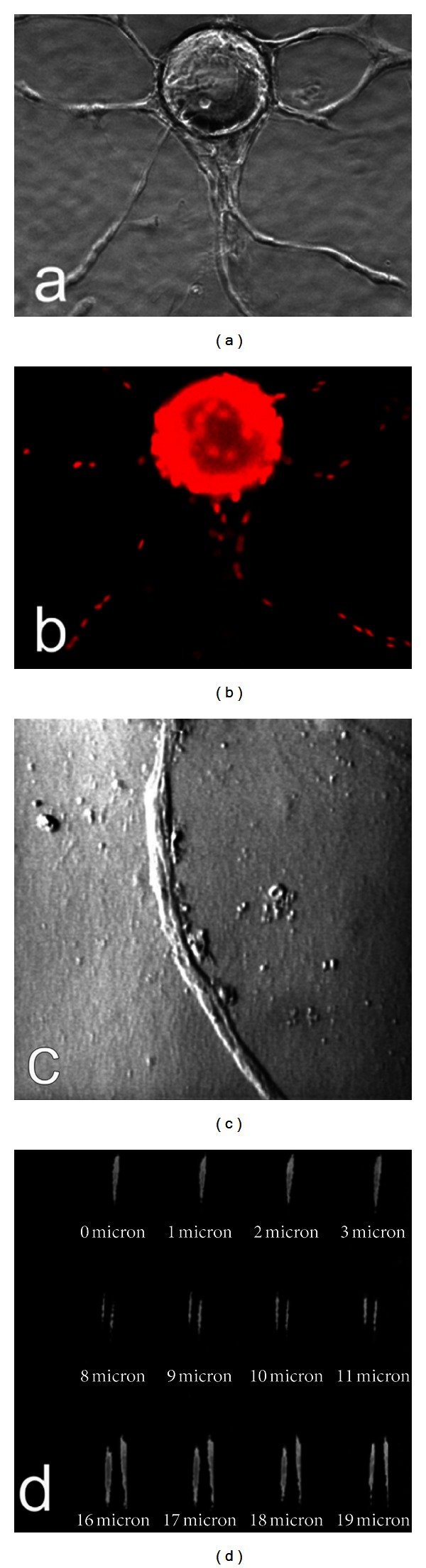
Presence of multiple cells in capillary tube-like structure by nuclei staining and presence of lumen demonstrated by confocal microscopic analysis. (a) HDMEC formed elongated and branched capillary sprouts in fibrin gel after 5 days in presence of VEGF (30 ng/mL) and bFGF (25 ng/mL). (b) The culture in (a) was fixed and stained with propidium iodide (PI) to reveal nuclei. (c) Phase-contrast photomicrograph of typical capillary tube-like structure formed by HDMEC in fibrin with VEGF (30 ng/mL) and bFGF (25 ng/mL). (d) Series of contiguous 1 micron thick tangential cross sections of the capillary tube-like structure, obtained by reflective confocal microscopy with computerized imaging, initially revealed the top of the tube (0–3 microns) and then the presence of a central lumen (8–19 microns) between two walls. (a) and (b) 100x and (c) and (d) 400x magnification.

To delineate why angiogenesis of early wound granulation tissue started by invading the fibrin clot as capillary sprouts, we studied the regulatory effect of fibrin microenvironment on sprout angiogenesis. In our modified microcarrier-based in vitro 3D human sprout angiogenesis system, when VEGF (30 ng/mL) and bFGF (25 ng/mL) were added to serum free fibrin gel containing EC-beads, HDMEC formed capillary sprouts, which projected from the surface of the EC-beads and invaded into the fibrin gel within 48 hr (Figure 3(b)). By 5 days the endothelial sprouts had elongated and in some cases formed branching capillary sprouts (Figure 3(c)). Local capillary networks formed by branching and fusion of capillary sprouts from the same bead (Figure 3(d)), and wild capillary networks formed by fusion of capillary sprouts from adjacent beads (Figures 3(e) and 3(f)). In contrast, without the addition of an angiogenesis factor, no significant HDMEC sprout formation occurred from the surface of EC-beads in fibrin gel, despite the presence of 20% normal human serum in the medium above the gels (Figure 3(a)). Fluorescence of cell nuclei staining clearly revealed that 5-day capillary sprouts (Figure 4(a)) were composed by multiple cells (Figure 4(b)). To demonstrate whether capillary tube-like structures (Figure 4(c)) had lumina, we used reflective confocal microscopic analysis. Computer-assisted sectioning clearly revealed the presence of a lumen (Figure 4(d)) in the capillary-like structure shown in Figure 4(a). As the tube was cut through by a series of 1 micron contiguous tangential cross sections, first the top and then the central lumen with two walls become visible (Figure 4(d)).

### 3.3. Fibrin and Collagen 3D Microenvironment Differentially Fine-Regulate Angiogenesis of HDEMC

**Figure 5:**
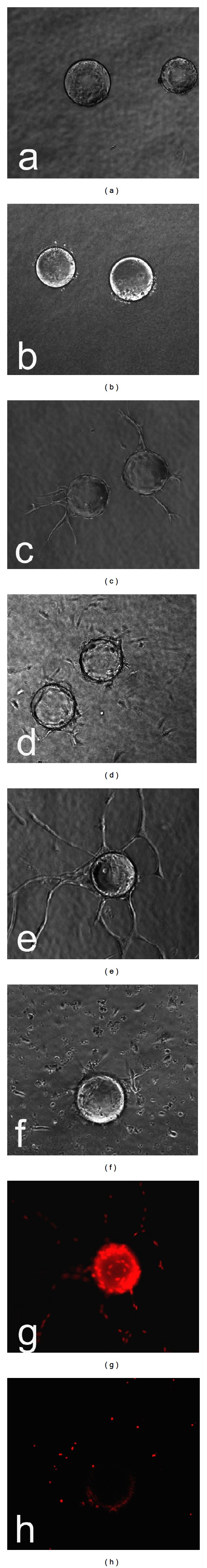
Fibrin gels (a, c, e, g) and collagen gels (b, d, f, h) differentially regulated HDMEC response to VEGF (30 ng/mL) and bFGF (25 ng/mL). Without the addition of angiogenesis factors, little or no sprout formation, invasion, or migration of HDMEC occurred either in fibrin gel (a) or in collagen gel (b). In the presence of VEGF (30 ng/mL) and bFGF (25 ng/mL), HDMEC formed capillary sprouts which invaded and migrated into fibrin gel within 48 hr (c). In contrast, in collagen gel in the presence of VEGF (30 ng/mL) and bFGF (25 ng/mL), HDMEC invaded and migrated into the gel individually without forming capillary sprouts (d). After 5 days, the capillary sprouts in fibrin elongated and branched (e). IF staining revealed that the capillary sprouts in fibrin were formed by multiple, lineal aligned cells (g). After 5 days, HDMEC randomly migrated into collagen gel without forming capillary sprouts (f). IF staining revealed that the nuclei were randomly distributed in collagen gel (h) (100x magnification).

 To determine whether ECM environment, fibrin and collagen in particular, regulates human sprout angiogenesis, a modified in vitro 3D angiogenesis system, which can differentiate sprouting angiogenesis from invasive migration, was used to compare the angiogenic response of HDMEC in 3D fibrin and collagen gels. EC-beads were embedded in fibrin gel or in collagen gel, with or without presence of VEGF (30 ng/mL) and bFGF (25 ng/mL) for 48 hr. In the absence of angiogenic stimulators, HDMEC remained on the surface of EC-beads and did not invade either fibrin gel (Figure 5(a)) or collagen gel (Figure 5(b)). In the presence of VEGF in fibrin gel, HDMEC formed capillary-like sprouts from the surface of EC-beads and invaded and migrated into the surrounding fibrin (Figure 5(c)). In contrast, when angiogenic factors were added to collagen gel, HDMEC invaded and migrated into the surrounding collagen as individual cells but did not form sprouts (Figure 5(d)). After 5 days, the capillary sprouts in fibrin elongated and fused into networks (Figure 5(e)), but in collagen individual HDMEC randomly migrated without capillary sprouts and networks (Figure 5(f)). Nucleus staining by PI confirmed that in fibrin the capillary tubes were composed of multiple cells (Figure 5(g)), but in collagen HDMEC randomly distributed in the gel (Figure 5(h)). 

### 3.4. Regulating Angiogenesis of HDMEC in Collagen by Modulating ECM Environment

**Figure 6:**
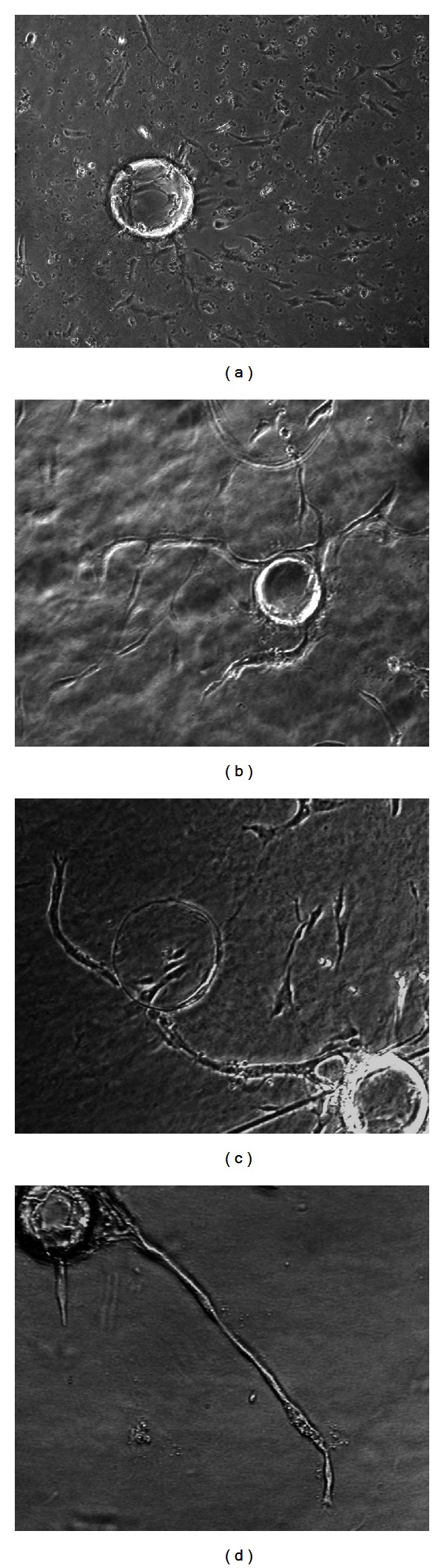
Fibrin facilitated sprout angiogenesis of HDMEC in collagen induced by angiogenic factors combo (30 ng/mL VEGF + 25 ng/mL bFGF) at 48 hr. Angiogenic factors stimulated HDMEC to invade and migrate into pure collagen gel individually without forming capillary sprouts (a). With the addition of 20% fibrin fibrils, by volume (b), or 30% fibrin fibrils, by volume (c), to collagen gel in the presence of angiogenic factors, sprout angiogenesis occurred together with individual cell invasion and migration. Angiogenic factors induced sprout angiogenesis of HDMEC in pure fibrin gel (d) (100x magnification).

 To determine if presence of fibrin in collagen gels will enable angiogenic factors to induce capillary sprouts in collagen gels, we embedded EC-beads in collagen admixed with fibrin and compared the angiogenic response to that in either pure fibrin or pure collagen gels. Combination of VEGF (30 ng/mL) and bFGF (25 ng/mL) induced individual cell invasion and migration in pure collagen gels (Figure 6(a)) and induced capillary sprouts in pure fibrin gels (Figure 6(d)) as previously observed. With addition of fibrin to collagen gel (Figure 6(b), 20% fibrin, and Figure 6(c), 30% fibrin), tube-like capillary sprout formation occurred together with individual cell invasion and migration. Thus the presence of fibrin appeared to be essential for HDMEC sprout angiogenesis induced by angiogenic factors.

### 3.5. Antagonists for Integrin Alpha v Beta 3 and Integrin Alpha 2 Beta 1 Synergistically Inhibit Sprout Angiogenesis of HDMEC in 3D Fibrin

**Figure 7:**
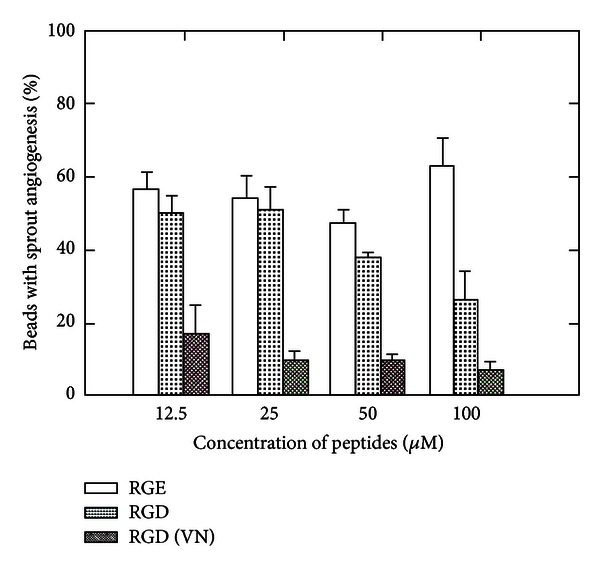
Effect of GPenGRGDSPCA peptides (RGD (VN)) on sprout angiogenesis of HDMEC in fibrin. HDMEC cultured on microcarrier beads were incubated with various doses of RGD (VN) (12.5 micro M–100 micro M) peptides at RT for 1 hr. The EC-beads were added to fibrinogen solution with presence of VEGF (30 ng/mL) and bFGF (25 ng/mL) and then polymerized with thrombin. RGD (VN) significantly inhibited sprout angiogenesis of HDMEC in fibrin compared to GRGESP(RGE) control peptides and nonspecific integrin antagonist cyclic RGD peptide (GRGDNP).

**Figure 8:**
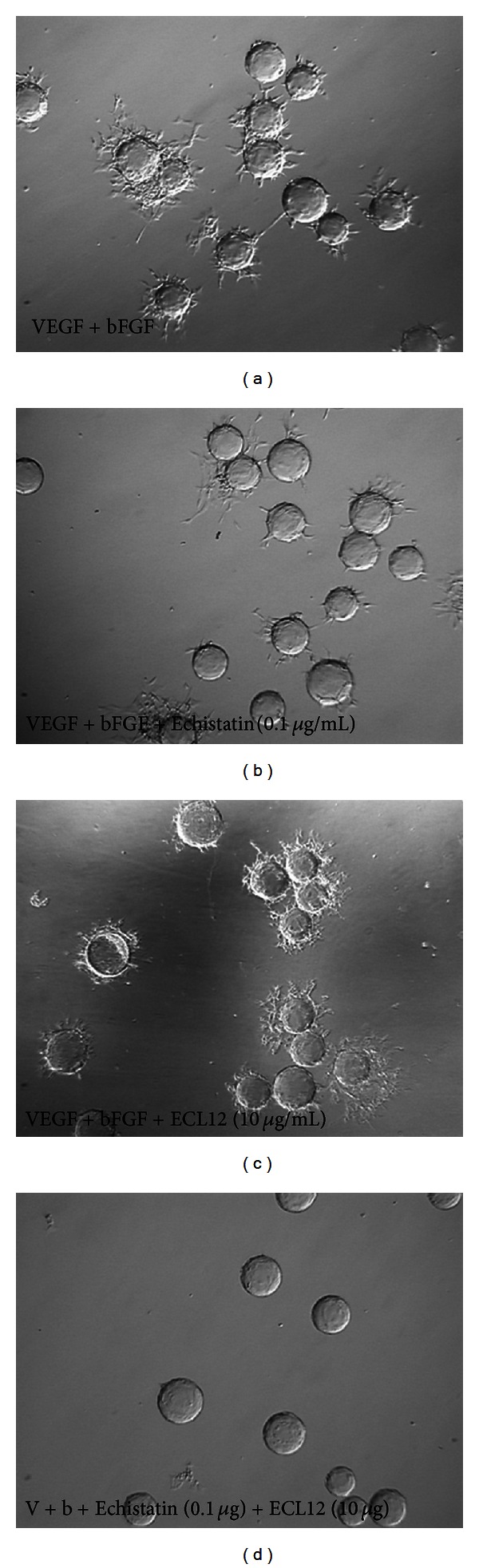
Integrin alpha v beta3 antagonist Echistatin and integrin alpha 2 beta 1 antagonist ECL12 differentially but synergistically inhibit sprout angiogenesis of HDMEC in fibrin. VEGF (30 ng/mL) and bFGF (25 ng/mL) induced sprout angiogenesis in fibrin (a). Echistatin at very low dose (0.1 microgram/mL) had mild inhibitory effect on sprout angiogenesis of HDMEC in fibrin (b). While disintegrin ECL12 had no inhibitory effect on sprout angiogenesis in vitro (c), it synergistically enhanced the inhibition of sprout angiogenesis by Echistatin (d).

 We recently demonstrated that integrin alpha v beta 3 is essential for sprout angiogenesis and integrin beta 3 expression is highly upregulated in fibrin rich but not in collagen rich matrix environment in vitro and in vivo [4, 13]. Using the modified in vitro sprout angiogenesis model, we demonstrated that GPenGRGDSPCA peptide (RGD (VN)), which is a specific antagonist for integrin alpha v beta 3, significantly blocked sprout angiogenesis of HDMEC in fibrin compared to the control RGE peptide (Figure 7). Echistatin (10 microgram/mL, 3 microgram/mL, 1 microgram/mL), a disintegrin specific for alpha v beta 3, dose dependently inhibits sprout angiogenesis of HDMEC in fibrin (data not shown). In contrast, ECL12, inhibitor to collagen receptor integrin alpha 3 beta 1, had no inhibitory effect on sprout angiogenesis of HDMEC in fibrin, even at a concentration 10 microgram/mL (Figure 8). At a concentration of 0.1 microgram/mL, Echistatin has minimal inhibitory effect on sprout angiogenesis of HDMEC in fibrin. However, the combination of 0.1 microgram/mL Echistatin and 10 microgram/mL ECL12 completely inhibited sprout angiogenesis of HDEMC in fibrin induced by VEGF (30 ng/mL) and bFGF (25 ng/mL). These data indicates that integrin alpha v beta 3 and integrin alpha 2 beta 1 differentially but synergistically inhibit sprout angiogenesis of HDMEC in 3D fibrin. It also suggests that collagen and fibrin differentially but synergistically regulate sprout angiogenesis.

## 4. Discussion

Alteration of fibrin and type I collagen matrices in the ECM microenvironment is a hallmark feature of wound clot and tumor stroma; however its role in the regulation of sprout angiogenesis is still poorly defined. Using in vitro 3D angiogenesis assay and in vivo wound healing model, we demonstrate in this study that robust angiogenic vessels invaded the fibrin clot of early wounds but matured and regressed as fibrin was replaced by type I collagen (Figure 2). These results are consistent with the finding that fibrin deposition is commonly observed in angiogenesis associated with wound healing and tumor growth. It has been reported that fibrin enhances angiogenesis of wound healing in vitro and in vivo [7, 8]. In contrast, type I collagen is a major component of normal dermis which has minimal angiogenesis activities. In our in vitro 3D angiogenesis assay, angiogenic stimulators induce endothelial cell migration but not sprout and capillary network formation in 3D type I collagen matrices (Figure 5). Interestingly once fibrin was added to type I collagen gel, the sprout angiogenesis is restored (Figure 6). Dvorak and colleagues also demonstrate that implanted fibrin gels themselves induce an angiogenic response in the subcutaneous space of guinea pigs in the absence of tumor cells or platelets, while type I collagen or agarose does not induce new blood vessel formation [8].

After tissue injury, type I collagen is removed with normal dermis and fibrinogen leaks from ligated blood vessels to form the fibrin clot, which fills the wound space [16–18]. Using in vivo wound healing model, we demonstrated that after a 3-day lag, endothelial cells migrate from the periphery of the wound and invade the fibrin clot as sprout angiogenesis to form nascent granulation tissue (Figure 2). Type I collagen accumulation begins as the granulation tissue matures, and in small excisional wounds, type I collagen replaces fibrin in the wound space by 7 days and form contracted wound scar in 10 days [19, 20]. We demonstrate here a substantial correlation between sprout angiogenesis and presence of fibrin matrices and the regression of sprout angiogenesis when fibrin is replaced by type I collagen. However, type I collagen supports sprout angiogenesis in the presence of fibrin fibrils. The regulatory mechanism of type I collagen in sprout angiogenesis is still not clear. Recently two teams of researchers demonstrated that an amino terminal peptide of angiocidin binds type I collagen and inhibits tumor growth and angiogenesis [21, 22]. 

Although fibrin is actively involved in regulating angiogenesis, we demonstrated that pure fibrin matrices themselves do not induce sprout angiogenesis without presence of angiogenic factors (Figure 5(a)). Roy et al. demonstrated that platelet-rich fibrin matrix improves wound angiogenesis via inducing vascular endothelial cell proliferation [23]. Lafleur and colleagues demonstrated that membrane-type-matrix metalloproteinases (MT-MMPs) are essential for endothelial angiogenesis in fibrin matrix [24]. It has been argued that if fibrin itself was sufficient to induce angiogenesis, it must perform at least two functions: (1) providing a three-dimensional matrix that supports cell migration and (2) expressing selective chemotactic and/or chemokinetic activity such that endothelial cells migrate into fibrin clot [8]. It is not surprising that fibrin might provide a three-dimensional matrix capable of supporting cell migration. However, it is unexpected that fibrin might directly induce cell migration without the presence of any soluble chemotactic factors. Greiling and Clark demonstrated that human dermal fibroblasts fail to migrate from a collagen matrix into a fibrin gel in the absence of platelet releasate or PDGF-BB [25]. Our in vitro data also demonstrated that there was no sprout angiogenesis of HDMEC in fibrin without presence of angiogenesis growth factors, such as VEGF and bFGF, in the matrix. Thus fibrin is essential to support sprout angiogenesis of microvascular endothelials induced by angiogenic stimulators in 3D environment.

Integrin alpha v beta 3 is the receptor for fibrin matrix. Expression of integrin alpha v beta 3 is one of the hallmark features of sprout angiogenesis. Accumulating evidence indicates that ECM microenvironment regulates cell functions through the integrin receptors. We recently demonstrated that fibrin and type I collagen differentially regulated integrin expression in human dermal microvascular endothelial cells (HDMEC) [4] and in human dermal fibroblasts [9]. In particular, fibrin, but not collagen, increased the expression of integrin alpha v beta 3 in HDMEC [4]. Since integrin alpha v beta 3 is the marker for sprout angiogenesis associated with wound healing and tumor growth [14, 25], this suggests that fibrin and collagen differentially regulate angiogenesis, in part, by altering endothelial cell integrin expression. Compared to highly regulated angiogenesis in wound healing, angiogenesis persists in solid tumor growth, as does the blood vessel leak of fibrinogen and resultant interstitial clotting [26]. Numerous studies indicated that integrin alpha v beta 3 has potential to be a novel target for cancer treatment [27–29]. Our results indicate that GPenGRGDSPCA peptide (RGD (VN)), which is a specific antagonist for integrin alpha v beta 3, significantly blocked sprout angiogenesis of HDMEC in fibrin induced by VEGF (Figure 7). Currently many new investigational cancer drugs based on RGD peptide are tested in clinical trials.

Integrin alpha 2 beta 1 is the integrin receptor for native collagens which mediate many important cellular functions, such as adhesion, migration, invasion, and contraction of collagen lattices. The role of integrin alpha 2 beta 1 in angiogenesis is controversial and is still not clearly defined [30]. Using in vitro 2D models, Senger and colleagues demonstrated that blocking antibodies for integrin alpha 2 beta 1 inhibit endothelial cell migration on type I collagen. In addition, they demonstrated that blocking antibodies to integrin alpha 2 beta 1 also potently inhibit VEGF induced angiogenesis using Matrigel transplant in mice skin [31]. In contrast, Zweers and colleagues found strong enhancement of angiogenesis in cutaneous wound and implanted sponges in alpha 2 null mice [32]. Furthermore, Zutter and colleagues demonstrated that poor expression of integrin alpha 2 beta 1 might play an essential role in cancer progression [33]. This is confirmed by Ramirez and colleagues in spontaneous mouse model of breast cancer showing alpha 2 beta 1 integrin suppresses metastasis [34]. Haidari and colleagues recently reported evidence that integrin alpha 2 beta 1 mediates tyrosine phosphorylation of vascular endothelial cadherin induced by invasive breast cancer cells, which is essential for transendothelial migration (TEM) of invasive cancer cells [35]. In our in vitro 3D angiogenesis assay, antagonists for integrin alpha 2 beta 1 demonstrate minimal inhibitory effect on sprout angiogenesis of HDMEC in 3D fibrin matrices. Interestingly, when combined with Echistatin, a disintegrin antagonist specific for integrin alpha v beta 3 and antagonists for integrin alpha 2 beta 1 synergistically inhibit sprout angiogenesis of HDMEC in fibrin. Taken together, these results suggest that fibrin and collagen differentially but synergistically regulate sprout angiogenesis through controlling integrin alpha v beta 3 and integrin alpha 2 beta 1 functions. 

This study indicated that growth factor-rich fibrin is essential to promote sprout angiogenesis. Fibrin and type I collagen 3D matrices differentially but synergistically regulate sprout angiogenesis in wound healing and solid tumors. Thus blocking both integrin alpha v beta 3 and integrin alpha 2 beta 1 might be a novel strategy to synergistically block sprout angiogenesis in solid tumors.
